# A simple clinical risk score (ABCDMP) for predicting mortality in patients with AECOPD and cardiovascular diseases

**DOI:** 10.1186/s12931-024-02704-6

**Published:** 2024-02-10

**Authors:** Jiarui Zhang, Qun Yi, Chen Zhou, Yuanming Luo, Hailong Wei, Huiqing Ge, Huiguo Liu, Jianchu Zhang, Xianhua Li, Xiufang Xie, Pinhua Pan, Mengqiu Yi, Lina Cheng, Hui Zhou, Liang Liu, Adila Aili, Yu Liu, Lige Peng, Jiaqi Pu, Haixia Zhou

**Affiliations:** 1 Department of Respiratory and Critical Care Medicine, West China Hospital, Sichuan University, Guo-xue-xiang 37#, Wuhou District, Chengdu, 610041 Sichuan Province China; 2 Sichuan Cancer Hospital, University of Electronic Science and Technology of China, Chengdu, Sichuan Province China; 3 West China School of Medicine, West China Hospital, Sichuan University, Chengdu, Sichuan Province China; 4 State Key Laboratory of Respiratory Disease, Guangzhou Medical University, Guangzhou, Guangdong Province China; 5 Department of Respiratory and Critical Care Medicine, People’s Hospital of Leshan, Leshan, Sichuan Province China; 6 Department of Respiratory and Critical Care Medicine, Sir Run Run Shaw Hospital, Zhejiang University School of Medicine, Hangzhou, Zhejiang Province China; 7 Department of Respiratory and Critical Care Medicine, Tongji Hospital, Tongji Medical College, Huazhong University of Science and Technology, Wuhan, Hubei Province China; 8 Department of Respiratory and Critical Care Medicine, Union Hospital, Tongji Medical College, Huazhong University of Science and Technology, Wuhan, Hubei Province China; 9 Department of Respiratory and Critical Care Medicine, the First People’s Hospital of Neijiang City, Neijiang, Sichuan Province China; 10 Department of Respiratory and Critical Care Medicine, Xiangya Hospital, Central South University, Changsha, Hunan Province China; 11 Department of Emergency, First People’s Hospital of Jiujiang, Jiu jiang, Jiangxi Province China; 12 Department of Respiratory and Critical Care Medicine, The Affiliated Hospital of Chengdu University, Chengdu, Sichuan Province China

**Keywords:** AECOPD, CVDs, Mortality, Risk score

## Abstract

**Background:**

The morbidity and mortality among hospital inpatients with AECOPD and CVDs remains unacceptably high. Currently, no risk score for predicting mortality has been specifically developed in patients with AECOPD and CVDs. We therefore aimed to derive and validate a simple clinical risk score to assess individuals’ risk of poor prognosis.

**Study design and methods:**

We evaluated inpatients with AECOPD and CVDs in a prospective, noninterventional, multicenter cohort study. We used multivariable logistic regression analysis to identify the independent prognostic risk factors and created a risk score model according to patients’ data from a derivation cohort. Discrimination was evaluated by the area under the receiver-operating characteristic curve (AUC), and calibration was assessed by the Hosmer–Lemeshow goodness-of-fit test. The model was validated and compared with the BAP-65, CURB-65, DECAF and NIVO models in a validation cohort.

**Results:**

We derived a combined risk score, the ABCDMP score, that included the following variables: age > 75 years, BUN > 7 mmol/L, consolidation, diastolic blood pressure ≤ 60 mmHg, mental status altered, and pulse > 109 beats/min. Discrimination (AUC 0.847, 95% CI, 0.805–0.890) and calibration (Hosmer‒Lemeshow statistic, *P* = 0.142) were good in the derivation cohort and similar in the validation cohort (AUC 0.811, 95% CI, 0.755–0.868). The ABCDMP score had significantly better predictivity for in-hospital mortality than the BAP-65, CURB-65, DECAF, and NIVO scores (all *P* < 0.001). Additionally, the new score also had moderate predictive performance for 3-year mortality and can be used to stratify patients into different management groups.

**Conclusions:**

The ABCDMP risk score could help predict mortality in AECOPD and CVDs patients and guide further clinical research on risk-based treatment.

**Clinical trial registration:**

Chinese Clinical Trail Registry NO.:ChiCTR2100044625; URL: http://www.chictr.org.cn/showproj.aspx?proj=121626.

**Supplementary Information:**

The online version contains supplementary material available at 10.1186/s12931-024-02704-6.

## Introduction

Chronic obstructive pulmonary disease (COPD) is characterized by persistent respiratory symptoms and progressive airflow obstruction and is associated with high rates of hospitalization and hospital length of stay (LOS) [1]. The most common causes include tobacco smoke, environmental exposures and genetic (inherited) risk [2]. According to the Global Initiative for Chronic Obstructive Lung Disease (GOLD) guideline, acute exacerbation of COPD (AECOPD) is defined as an event characterized by increased dyspnea and/or cough and sputum that worsens in < 14 days, which may be accompanied by tachypnea and/or tachycardia and is always involved in more severe health conditions and more radical treatment [3–5]. The mortality related to AECOPD rises from 4.5 to 25.4% with increased severity of AECOPD [6–8]. AECOPD is now one of the top three causes of death worldwide, and it is estimated that there will be over 5.4 million annual deaths from AECOPD and related conditions by 2060 [3, 9].

Cardiovascular diseases (CVDs) are a leading cause of silent massive heart attacks, which places a huge burden on health care systems [10]. Most prior studies have revealed that CVDs are a common complication and contribute significantly to both morbidity and mortality in COPD [11, 12]. Patients with COPD are at higher risk of developing CVDs when compared with patients without COPD [13]. In addition, the presence of CVDs increases the risk of frequent exacerbations of COPD [14]. Cardiovascular comorbidities can significantly affect the outcomes of patients with AECOPD, leading to disease progression, worsening clinical outcomes, longer length of stay and even mortality [15, 16].

Considering the high prevalence of CVDs among patients with AECOPD, risk evaluation and prediction of mortality would be important to improve prognosis. Several prognostic models have been derived and validated for risk stratification in AECOPD patients, such as the BAP-65 (blood urea nitrogen ≥ 25 mg/dl, altered mental status, pulse > 109 beats/min, age > 65 years) [17], CURB-65 (confusion of new onset, blood urea nitrogen (BUN) > 7 mmol/L (19 mg/dL), respiratory ⩾30 breaths·min − 1; systolic blood pressure < 90 mmHg or diastolic blood pressure ≤ 60 mmHg , age ⩾65 years) [18], DECAF (dyspnoea, eosinopenia, consolidation, acidaemia and atrial fibrillation) [19, 20], or Noninvasive Ventilation Outcomes (NIVO) score (chest radiograph consolidation, Glasgow Coma Scale ≤ 14, atrial fibrillation, pH < 7.25, time to acidaemia > 12 h, eMRCD5a, eMRCD5b) [8]. However, these risk models predict in-hospital mortality in all AECOPD patients regardless of whether CVDs are present but were not designed for use in this population, and their performance and applicability were less strong in patients with AECOPD and CVDs. Hence, a novel and reliable prediction tool that can accurately risk stratify patients with AECOPD and CVDs is needed.

The primary aim of the present study was to derive and validate a simple clinical risk stratification score, identifying patients with either a high or low probability of short-term adverse outcomes in inpatients with AECOPD and CVDs from a prospective multicenter cohort study in China. We compared the performance of this novel score with that of the BAP-65, CURB-65, DECAF and NIVO models. In addition, we also evaluated the predictive performance of the risk score for long-term mortality in patients with AECOPD and CVDs.

## Methods

### Study design and patients

The MAGNET AECOPD (MAnaGement aNd advErse ouTcomes in inpatients with acute exacerbation of COPD) Registry study was a prospective, noninterventional, multicenter cohort study enrolling consecutive inpatients with AECOPD. Data collection was performed by ten tertiary hospitals in China between September 2017 and July 2021. In the present study, patients with objectively diagnosed AECOPD and CVDs were included from the AECOPD database. Exclusion criteria included duration of stay < 48 h and data missed. For patient inclusion, we screened the database for patients with AECOPD and CVDs first, and then the patient records were reviewed to determine whether they were eligible for inclusion. AECOPD is defined as periodic deterioration of respiratory symptoms, resulting in the need for urgent care or hospitalization and a decline in quality of life [21]. CVDs included coronary heart disease, heart failure, heart valve problems, arrhythmia and stroke, according to the American Heart Association. The study population was divided into a derivation cohort and a validation cohort, including patients from West China Hospital, Guangzhou Medical University, Xiangya Hospital, Tongji and Union Hospitals of Huazhong University of Science and Technology, People’s Hospital of Leshan, Sir Run Run Shaw Hospital, the First People’s Hospital of Neijiang City, the First People’s Hospital of Jiujiang, and the Affiliated Hospital of Chengdu University. All patients were treated according to clinical practice standards in the institutions. The study protocol was approved by the institutional review boards of the ten academic medical centers that participated. Written informed consent was obtained from all patients.

### Data collection

For this study, clinical data of participants were collected with a standardized case report form, including baseline demographics, comorbidities, vital signs, laboratory and radiologic findings, and treatments, as described previously [22]. The data collectors received in-depth training, and all data were checked by two investigators (JZ and CZ) to ensure the reliability of the information. In addition, prognostic variables comprising the clinical scores were determined to calculate the total scores of BAP-65, CURB-65, DECAF, and NIVO for all patients. In the MAGNET AECOPD Registry study, the enrolled individuals received follow-up for 3 years by telephone, outpatient visits, or rehospitalization.

### Study outcomes

The main outcome was all-cause in-hospital mortality. The secondary outcome was mortality at 3 years. All clinical outcomes were adjudicated by the independent clinical event committee composed of three experienced physicians.

### Statistical analysis

Categorical variables are reported as frequencies and percentages, and significant differences were analysed by using the chi-squared test. Continuous variables are reported as the median and interquartile range, and significant differences were analysed using Wilcoxon’s rank-sum test. Multivariable logistic regression analysis was used to identify independent risk factors for in-hospital mortality in the derivation cohort. The independent risk factors were ascribed relative weighting according to their regression coefficient and constituted the new predictive score. Discrimination was evaluated by the area under the receiver operating characteristic curve (AUC), and calibration was assessed by the Hosmer–Lemeshow goodness-of-fit test. Given that the BAP-65, CURB-65, DECAF, and NIVO scores have been previously reported to be used for risk stratification in AECOPD patients [8, 17–19], we also performed an exploratory analysis to investigate how the new predictive score compares against these scores. Kaplan‒Meier curves were computed to present the survival rates of patients with AECOPD and CVDs after dividing patients into 3 groups (low, medium, and high risk) according to the new score. All statistical analyses were conducted using SPSS version 25.0 (IBM, New York, United States). They were two-tailed, and *P* values < 0.05 indicated a statistically significant difference.

## Results

### Study population

**Fig. 1:**
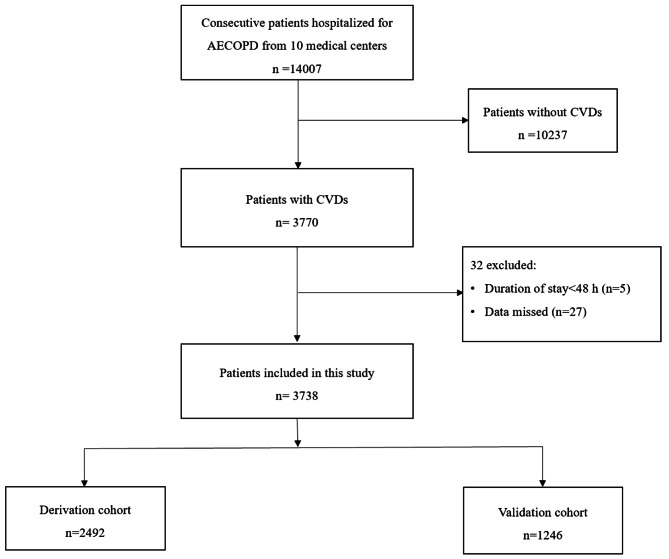
The flowchart of patient enrollment for the derivation and validation cohorts Abbreviations: AECOPD = Acute exacerbation of chronic obstructive pulmonary disease; CVDs = cardiovascular diseases

Among the 14,007 patients enrolled in the registration study for AECOPD, 3738 were included in this analysis. The main reasons for exclusion were as follows: (1) patients without CVDs (*n* = 10,237); (2) hospitalization for less than 2 days (*n* = 5); and (3) data missed (*n* = 27). Among them, 122 (3.26%) died during hospitalization, 241 (6.45%) patients had used invasive mechanical ventilation during their hospital stay, and 474 (12.68%) patients had been treated in the intensive care unit (ICU) (Fig. 1).

The baseline characteristics of the derivation cohort are presented in Table 1. A total of 2492 subjects entered the final analysis, in which 84 subjects died during hospitalization, with an overall mortality of 3.37%. Compared with subjects without mortality, the patients who died in hospital were older, with faster radial pulse as well as lower diastolic blood pressure (DBP), and had a higher frequency of altered mental status (all *P* < 0.05). In terms of laboratory tests, patients with overall mortality had higher rates of anemia and acidaemia as well as higher white blood cell counts, blood urea nitrogen, N-terminal pro-B–type natriuretic peptide (NT-pro BNP), and troponin T (all *P* < 0.05). Regarding radiographic findings, consolidation and pleural effusion were more frequently seen in patients with overall mortality.

**Table 1:** Description of derivation and validation cohorts

Characteristics	Derivation cohort			Validation cohort		
	With overall mortality, n (%) or median (IQR)	Without overall mortality, % (n) or median (IQR)	*P*-Value	With overall mortality, n (%) or median (IQR)	Without overall mortality, n (%) or median (IQR)	*P*-Value
Total, n	84	2408		38	1208	
Age > 75 y	67(79.8)	1415(58.8)	< 0.001	32(84.2)	666(55.1)	< 0.001
Gender			0.252			0.543
Male	59(70.2)	1823(75.7)		31(81.6)	935(77.4)	
Female	25(29.8)	585(24.3)		7(18.4)	273(22.6)	
Smoking (current or past)	46(54.8)	1422(59.1)	0.424	17(44.7)	691(57.2)	0.125
Diabetes	21(25.0)	450(18.7)	0.146	13(34.2)	230(19.0)	0.020
CVDs						
Coronary Heart Disease	33(39.3)	947(39.3)	0.994	6(15.8)	558(46.2)	< 0.001
Heart failure	49(58.3)	1031(42.8)	0.005	25(65.8)	493(40.8)	0.002
Heart Valve Problems	3(3.6)	188(7.8)	0.208	3(7.9)	55(4.6)	0.417
Arrhythmia	38(45.2)	740(30.7)	0.005	16(42.1)	382(31.6)	0.172
Stroke	22(26.2)	533(22.1)	0.380	14(36.8)	243(20.1)	0.012
Pulse > 109 beats per min	20(23.8)	257(10.7)	< 0.001	8(21.1)	129(10.7)	0.044
SBP < 90 mm Hg	3(3.6)	33(1.4)	0.119	1(2.6)	22(1.8)	0.513
DBP ≤ 60 mm Hg	24(28.6)	219(9.1)	< 0.001	11(28.9)	111(9.2)	< 0.001
Respirations ≥ 30 per min	0(0)	45(1.9)	0.403	2(5.3)	23(1.9)	0.175
Altered mental status	36(42.9)	142(5.9)	< 0.001	9(23.7)	60(5.0)	< 0.001
Anemia	58(69.0)	1240(51.7)	0.002	29(78.4)	648(53.9)	0.003
WBC > 10 × 103 mm-3	34(40.5)	632(26.3)	0.004	18(47.4)	317(26.4)	0.004
ESOR < 2%	67(81.7)	1719(72.3)	0.062	32(84.2)	830(69.6)	0.053
PH < 7.3	7(8.3)	60(2.5)	0.001	3(7.9)	35(2.9)	0.106
Serum BUN > 7 mmol/L	60(71.4)	1022(42.4)	< 0.001	32(84.2)	496(41.1)	< 0.001
NT-pro BNP > 1000 pg/ml	63(75.9)	1084(51.6)	< 0.001	27(79.4)	494(46.2)	< 0.001
Troponin T > 200 ng/L	12(15.0)	83(4.4)	< 0.001	5(13.5)	27(2.8)	< 0.001
Consolidation	87(71.3)	1242(34.2)	< 0.001	25(65.8)	445(36.8)	< 0.001
Pleural effusion	45(53.6)	697(28.9)	< 0.001	12(31.6)	388(32.1)	0.944
BAP-65	3(3–4)	2(2–3)	< 0.001	3(3–4)	2(2–3)	< 0.001
CURB-65	2(2–3)	1(1–2)	< 0.001	2(2–3)	1(1–2)	< 0.001
DECAF	3(3–4)	2(2–3)	< 0.001	3(3–4)	2(2–3)	0.003
NIVO	4(3–5)	3(2–4)	< 0.001	4(3–5)	3(2–4)	0.019

Data are presented as the number of patients (%); mean ± standard deviation; median (interquartile range)

Abbreviations: CVDs = cardiovascular diseases; SBP = systolic blood pressure; DBP = diastolic blood pressure; WBC = white blood cell; EOSR = eosinophil ratio; BUN = blood urea nitrogen; NT-pro BNP = N-terminal pro-B-type natriuretic peptide;

Note: Anemia: hemoglobin is less than 12 g/L in females and hemoglobin is less than 13 g/L in males

### Predictors of in-hospital overall mortality

The results of univariable analysis for all potential predictors are shown in Table 2. Multivariable predictors of in-hospital mortality included age > 75 years (OR, 1.914; 95% CI, 1.080–3.392), pulse > 109 beats per minute (OR, 2.465; 95% CI, 1.394–4.358), DBP ≤ 60 mmHg (OR, 2.711; 95% CI, 1.567–4.691), altered mental status (OR, 6.269; 95% CI, 3.815–10.303), serum BUN > 7 mmol/L (OR, 2.280; 95% CI, 1.371–3.794) and consolidation (OR, 3.627; 95% CI, 2.161–6.088) (Table 2).

**Table 2:** Univariable and multivariable predictors of in-hospital mortality in the derivation Cohort

	OR (95%CI)	*P*	OR (95%CI)	*P*
	Univariable analysis		Multivariable analysis	
Age > 75 y	2.766(1.614–4.739)	< 0.001	1.914(1.080–3.392)	0.026
Pulse > 109 beats per min	2.616(1.557–4.393)	< 0.001	2.465(1.394–4.358)	0.002
DBP ≤ 60 mm Hg	3.998(2.441–6.548)	< 0.001	2.711(1.567–4.691)	< 0.001
Altered mental status	11.968(1.302–3.329)	< 0.001	6.269(3.815–10.303)	< 0.001
Anemia	2.081 (1.302–3.329)	0.002	…	…
WBC > 10 × 103 mm-3	1.901(1.218–2.967)	0.005	…	…
PH < 7.3	3.558(1.575–8.038)	0.002	…	…
Serum BUN > 7 mmol/L	3.390(2.097–5.480)	< 0.001	2.280(1.371–3.794)	0.002
NT-pro BNP > 1000 pg/ml	2.958(1.776–4.928)	< 0.001	…	…
Troponin T > 200 ng/L	3.799(1.980–7.292)	< 0.001	…	…
Consolidation	5.750(3.510–9.422)	< 0.001	3.627(2.161–6.088)	< 0.001
Pleural effusion	2.832(1.828–4.388)	< 0.001	…	…

Abbreviations: DBP = diastolic blood pressure; WBC = white blood cell; EOSR = eosinophil ratio; BUN = blood urea nitrogen; NT-pro BNP = N-terminal pro-B-type natriuretic peptide;

Note: Anemia: hemoglobin is less than 12 g/L in females and hemoglobin is less than 13 g/L in males

### Risk score construction

We combined these independent risk factors into the new simple ABCDMP score (Table 3): A: age > 75 years (1 point); B: BUN > 7 mmol/L (1 points); C: consolidation (2 points); D: DBP ≤ 60 mmHg (2 points); M: mental status altered (3 points); and P: pulse > 109 beats per minute (1 point). The total score ranged from 0 to 10 points. Patients with higher risk scores were at greater risk for in-hospital mortality; the OR for mortality per 1-point increase in the score was 1.863 (95% CI, 1.685–2.060; *P* < 0.001).

**Table 3:** ABCDMP score for poor outcome in patients with AECOPD and CVDs

Acronym	Risk Factor	Points
A	Age > 75 y (1 point)	1
B	BUN > 7 mmol/L (1points)	1
C	Consolidation (2 points)	2
D	DBP ≤ 60 mmHg (2 points)	2
M	Mental status (3 points)	3
*P*	Pulse > 109 beats per min (1 points)	1
	Total points	0–10
	AUC	95% CI
ABCDMP	0.847	0.805–0.890

Abbreviations: AECOPD = acute exacerbation of chronic obstructive pulmonary disease; CVDs = cardiovascular diseases; DBP = diastolic blood pressure

### Risk score performance and internal validation

**Fig. 2:**
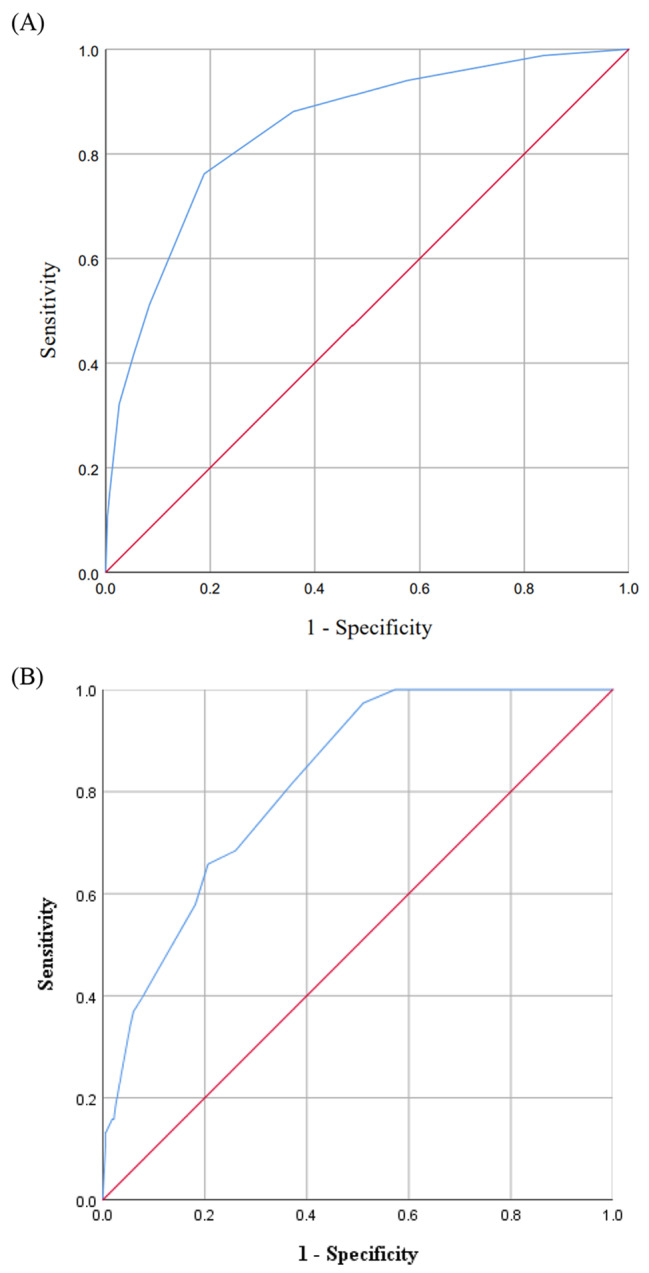
**A**, **B**, Receiver operating characteristic curves for the ABCDMP score in predicting in-hospital mortality: (**A**) derivation cohort and (**B**) validation cohort

The score showed good discrimination with an AUC of 0.847 (95% CI, 0.805–0.890) (Fig. 2A) and good calibration (*P* = 0.142). A total of 1246 subjects were enrolled in the validation cohort, and there was no significant difference in baseline characteristics between the derivation cohort and the validation cohort (Supplementary Table S1). When applied to the validation cohort, the score also showed good discrimination with an AUC of 0.811 (95% CI, 0.755–0.868) (Fig. 2B). Patients were classified into three risk categories for mortality based on total point scores: low risk (score, 0–1 point), medium risk (score, 2–4 points), and high risk (score, > 4 points). The mortality rate during hospitalization in patients with AECOPD and CVDs determined by the novel risk score, ABCDMP, is shown in Table 4. The mortality rate increased with the addition of each risk factor or risk category from the derivation cohort and the validation cohort.

**Table 4:** Survival Rate during hospitalization in the derivation and validation cohorts according to the risk score

	Derivation cohort(*n* = 2492)		Validation cohort(*n* = 1246)	
	No. (%)	Mortality Rate (%)	No. (%)	Mortality Rate (%)
ABCDMP score				
0	394(15.81)	0.25	212(17.01)	0.00
1	631(25.32)	0.63	289(23.19)	0.00
2	528(21.19)	0.95	257(20.63)	2.72
3	421(16.89)	2.38	225(18.06)	3.11
4	273(10.96)	7.69	154(12.36)	6.49
5	81(3.25)	9.88	49(3.93)	8.16
6	75(3.01)	10.67	29(2.33)	13.79
7	57(2.29)	24.56	19(1.52)	5.26
8	15(0.60)	26.67	2(0.16)	50.00
9	17(0.68)	52.94	8(0.64)	37.50
10	0(0.00)	…	2(0.16)	50.00
Risk category				
Low (0–1)	1025(41.13)	0.49	501(40.21)	0.00
Medium (2–4)	1222(49.04)	2.95	636(51.04)	3.77
High (> 4)	245(9.83)	17.55	109(8.75)	12.84
AUC (95%CI)	0.847(0.805–0.890)		0.811(0.755–0.868)	

### Comparison of the ABCDMP score with the BAP-65, CURB-65, DECAF, and NIVO scores

Based on the ROC curve analysis, the ABCDMP score had significantly better predictivity for in-hospital mortality than the BAP-65, CURB-65, DECAF, and NIVO scores in the derivation cohort (AUC BAP-65, 0.762; 95% CI, 0.708–0.816; *P* < 0.001 vs. ABCDMP; AUC CURB-65, 0.759; 95% CI, 0.704–0.815; *P* < 0.001 vs. ABCDMP; AUC DECAF, 0.685; 95% CI, 0.631–0.738; *P* < 0.001 vs. ABCDMP; AUC NIVO, 0.690; 95% CI, 0.631–0.748; *P* < 0.001 vs. ABCDMP) (Supplementary Figure S1A). In the validation cohort, the ABCDMP score also had significantly better predictivity for in-hospital mortality than the BAP-65, CURB-65, DECAF, and NIVO scores (AUC BAP-65, 0.742; 95% CI, 0.659–0.825; *P* < 0.001 vs. ABCDMP; AUC CURB-65, 0.775; 95% CI, 0.700–0.850; *P* < 0.001 vs. ABCDMP; AUC DECAF, 0.629; 95% CI, 0.538–0.720; *P* < 0.001 vs. ABCDMP; AUC NIVO, 0.619; 95% CI, 0.533–0.706; *P* < 0.001 vs. ABCDMP) (Supplementary Figure S1B).

### Validation of the ABCDMP score for long-term mortality

**Fig. 3:**
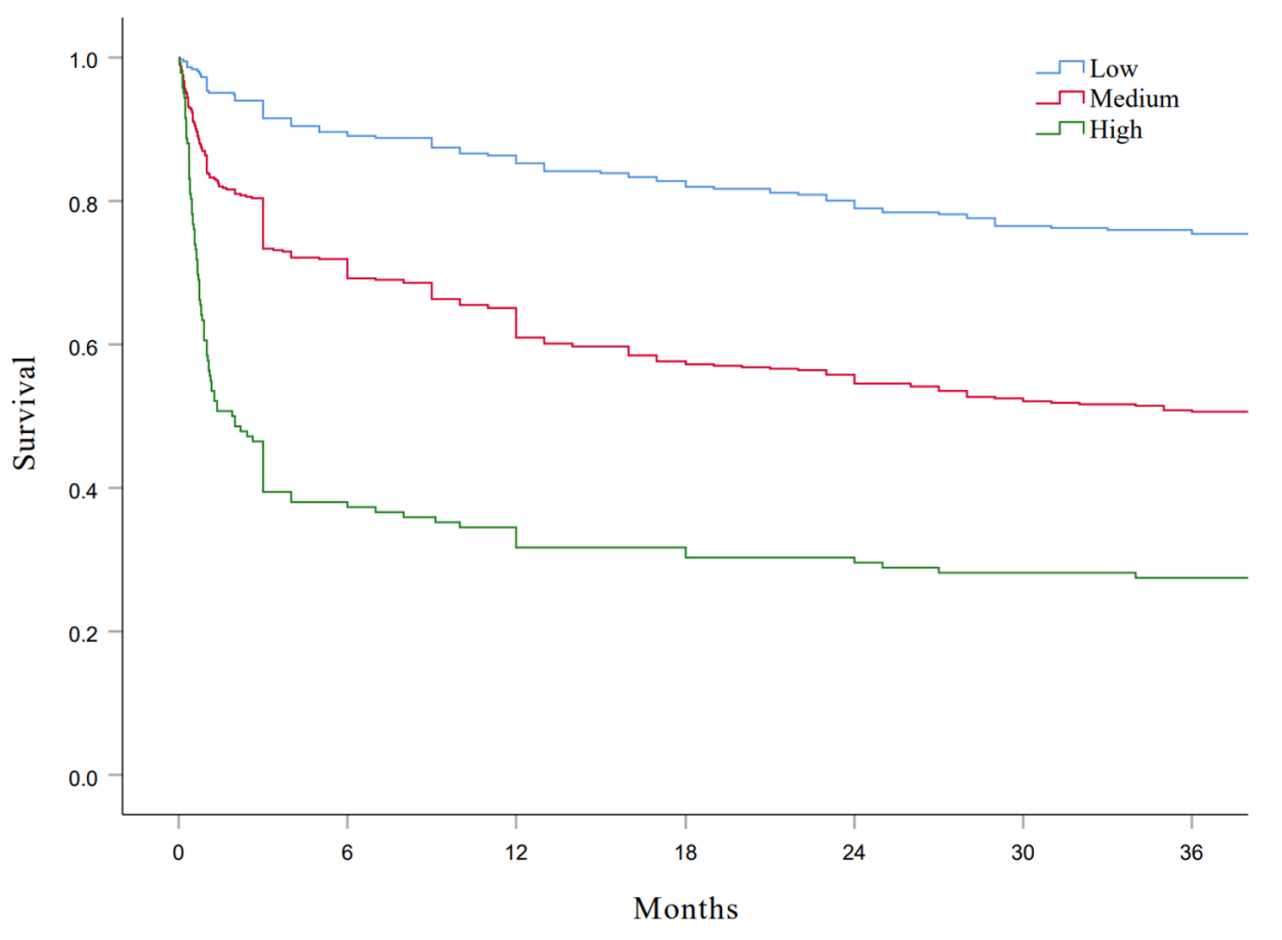
Kaplan-Meier curves for risk categories according to the ABCDMP score. Patients were divided into three groups: low (0–1 points), medium (2–4 points), and high risk (> 4 points)

We validated the ABCDMP score in the overall cohort of patients with available long-term follow-up data. The predictive performance of the BCDMP score as judged with AUC-ROC was 0.703 (95% CI, 0.670–0.735; *p* < 0.001) for mortality at 3 years (Supplementary Figure S2). The predictive value of risk categories of the ABCDMP score on mortality during the 3-year follow-up was further investigated by time-to-event analysis (Fig. 3). Kaplan‒Meier curves for risk categories showed an increased risk of mortality across the three groups (log-rank test *P* < 0.001).

## Discussion

In this large multicenter cohort, we identified six easily available factors that are associated with in-hospital mortality and derived the new ABCDMP score in patients with AECOPD and CVDs. To our knowledge, this was the first clinical score to assess individuals’ risk of poor prognosis in patients with AECOPD and CVDs. The clinical score can be calculated early after patient presentation and performs well in predicting mortality in patients with AECOPD and CVDs. It is superior to the existing optimal risk scores for predicting adverse outcomes in AECOPD. Additionally, the new score had moderate predictive performance for long-term mortality and could discriminate between patients at low, medium and high risk of mortality in patients with AECOPD and CVDs.

CVDs risk in COPD is very high compared with the general population due to lung hyperinflation, pulmonary hypertension and systemic inflammation [13, 23]. In a large cohort of patients with COPD admitted to a Veterans Administration hospital, the prevalence of coronary artery disease was 33.6%, significantly higher than the 27.1% prevalence seen in a matched cohort without COPD [24]. In a large and possibly most conclusive systematic review, Chen et al. found a nearly 2.5-fold increased risk of cardiovascular disease overall and a two- to five-fold higher risk of major cardiovascular disease types (ischemic heart disease, cardiac dysrhythmia, heart failure, diseases of the pulmonary circulation, and arterial diseases) in patients with COPD [15]. Hospitalized acute exacerbations are associated with mortality of cardiovascular events in COPD. Using a health insurance research database in Taiwan, Wang et al. found that the 90-day mortality rates of acute myocardial infarction and ischemic stroke in COPD patients without acute exacerbations were significantly lower than those in patients with hospitalized acute exacerbations (33.9% vs. 44.6% and 13.8% vs. 20.3%; all *p* < 0.001) [25]. Given the high morbidity and mortality, it is necessary to establish valid tools for the risk stratification of patients with AECOPD and CVDs.

In the multivariable analysis, we found that independent risk factors for in-hospital mortality in patients with AECOPD and CVDs were age > 75 years, pulse > 109 beats per minute, DBP ≤ 60 mmHg, altered mental status, BUN > 7 mmol/L and consolidation. These factors have been established as being associated with outcome in AECOPD/CVDs in previous studies [19, 26–30]. For instance, a study by Byrd et al. showed that a higher pulse rate was more linearly related to increases in all-cause mortality and cardiovascular events in patients with COPD [27]. Similar to this former study, we also observed a significant reduction in DBP in patients who died during hospitalization. Lower DBP on admission was reported to be associated with increased mortality and excess cardiovascular events in previous studies [27, 29]. Serum BUN > 7 mmol/L was also proven to be an independent risk factor for in-hospital mortality in patients with AECOPD and CVDs, and the same results were shown in our study as well [26, 28, 30].

As previously highlighted, previous studies have proposed several predictive tools for mortality in patients with AECOPD, such as the BAP-65 score [17], the CURB-65 score [18], the DECAF score [19, 20], and the NIVO score. BAP-65 is a disease-specific severity-of-illness score for AECOPD, which was designed to use only information that is generally available to physicians at the time of patient presentation. CURB-65 is simple to apply but was designed for use in patients with pneumonia. DECAF is a clinical prediction tool, incorporating indices routinely available at the time of hospital admission and can stratify patients hospitalized with AECOPD into clinically relevant risk. The NIVO score allows for accurate risk stratification of patients admitted to the hospital with AECOPD complicated by acidaemia and acute hypercapnic respiratory failure who required assisted ventilation. All these scoring systems were derived from large cohorts and showed good predictive values. However, the performance of the scores in predicting in-hospital mortality of patients with AECOPD and CVDs has not been commonly reported. In our study, we found that the discriminative power of the predictive risk scores in predicting in-hospital mortality of patients with AECOPD and CVDs was acceptable but dissatisfactory after ROC curve analysis (AUC, 0.619–0.775).

In our study, ROC curve analysis showed an excellent discriminate power of our ABCDMP model (AUC = 0.847, 95% CI, 0.805–0.890; *P* < 0.001), after which a validation of our ABCDMP model was performed using a multicenter cohort, revealing the validated AUC of our ABCDMP of 0.811. No in-hospital mortality occurred among patients with AECOPD and CVDs who scored < 2 with our ABCDMP, while the in-hospital mortality of patients who scored > 4 with our ABCDMP was 12.84% during hospitalization. Moreover, the AUC of our ABCDMP score was statistically greater than those of the BAP-65, CURB-65, DECAF, and NIVO scores (AUC BAP-65, 0.742; 95% CI, 0.659–0.825; *P* < 0.001 vs. ABCDMP; AUC CURB-65, 0.775; 95% CI, 0.700–0.850; *P* < 0.001 vs. ABCDMP; AUC DECAF, 0.629; 95% CI, 0.538–0.720; *P* < 0.001 vs. ABCDMP; AUC NIVO, 0.619; 95% CI, 0.533–0.706; *P* < 0.001 vs. ABCDMP) after the Z test, which suggested that the discriminatory value of our risk score for predicting in-hospital mortality in patients with AECOPD and CVDs was significantly better. Although this new risk score was originally developed for the short-term prediction of mortality, the score also showed moderate predictive performance for 3-year mortality in patients with AECOPD and CVDs (AUC: 0.703, 95% CI, 0.670–0.735; *p* < 0.001). It can be used to stratify patients admitted to the hospital with AECOPD and CVDs into different management groups (*P* < 0.001).

The ABCDMP score might also be helpful for clinical decision-making regarding the selection of management strategies. Patients at low risk may receive outpatient treatment or discharge them early, which could ease their financial burden of hospitalization and reduce the unnecessary waste of medical resources. Those at high risk can be considered for early interventions and escalation of care. Finally, patients with medium-risk scores can be managed with regular reassessment of risk. However, the calculation of a risk score cannot be the only variable determining such far-reaching decisions, which must take into account many other individual aspects, such as the patient’s wishes, comorbidities, and economic situation. Nevertheless, the ABCDMP score might provide valuable assistance.

### Strengths and limitations

This study is the first attempt to derive and validate a clinical prognostic score among inpatients with AECOPD and CVDs. It has several strengths, including the enrollment of patients from diverse hospitals and a near complete prospective data collection. In addition, the ABCDMP score is easy to calculate and apply in clinical practice and allows for good identification of patients at risk for in-hospital mortality. Nevertheless, our study had limitations. First, this was a secondary analysis of a prospective cohort of patients with AECOPD and CVDs, and we could not reach all baseline characteristics. Fortunately, the proportion of excluded patients was small, and the impact on our results can be neglected. Second, our sample included inpatients with AECOPD and CVDs; thus, our results may not be generalizable to outpatients or those with hospital at home.

## Conclusion

The ABCDMP risk score is a simple, user-friendly score to estimate the risk of in-hospital mortality in patients with AECOPD and CVDs, which exhibits better performance compared with existing scoring systems in the AECOPD setting. It may serve as a tool for risk stratification of AECOPD and CVDs patients and may thus be helpful for clinical decision-making and for the design of future clinical trials.

### Electronic supplementary material

Below is the link to the electronic supplementary material.

Supplementary Material 1

## Data Availability

The datasets used and analyzed during the present study are available from the corresponding author on reasonable request.

## Abbreviations

COPD: Chronic obstructive pulmonary disease
LOS: Length of stay
GOLD: Global Initiative for Chronic Obstructive Lung Disease
AECOPD: Acute exacerbation of chronic obstructive pulmonary disease
CVDs: Cardiovascular diseases
BUN: Blood urea nitrogen
NIVO: Noninvasive Ventilation Outcomes
MAGNET AECOPD: MAnaGement aNd advErse ouTcomes in inpatients with acute exacerbation of COPD) Registry
AUC: Area under the receiver operating characteristic curve
ICU: Intensive care unit
DBP: Diastolic blood pressure
NT-proBNP: N-terminal pro-B-type natriuretic peptide
OR: Odds ratio
95% CI: 95% confident interval
