# Supplementary material for: A simple clinical risk score (ABCDMP) for predicting mortality in patients with AECOPD and cardiovascular diseases
Source: Respir Res. 2024 Feb 10;25:89. doi: 10.1186/s12931-024-02704-6 (PMC10858518; doi:10.1186/s12931-024-02704-6)

Supplementary Table S1 Characteristics of derivation and validation cohorts.

| Characteristics | All, n (%) or median (IQR) | Derivation cohort n (%) or median (IQR) | Validation cohort,  n (%) or median (IQR) | p-Value |
| --- | --- | --- | --- | --- |
| Total, n | 3738 | 2492 | 1246 |  |
| Age >75 y | 2180(58.3) | 1482(59.5) | 698(56.0) | 0.044 |
| Gender |  |  |  | 0.175 |
| Male | 2848(76.2) | 1882(75.5) | 966(77.5) |  |
| Female | 890(23.8) | 610(24.5) | 280(22.5) |  |
| Smoking (current or past) | 2176(58.3) | 1468(59.0) | 708(56.9) | 0.217 |
| Diabetes | 714(19.1) | 471(18.9) | 243(19.5) | 0.659 |
| CVDs |  |  |  |  |
| Coronary Heart Disease | 1544(41.3) | 980(39.3) | 564(45.3) | 0.001 |
| Heart failure | 1598(42.8) | 1080(43.3) | 518(41.6) | 0.304 |
| Heart Valve Problems | 249(6.7) | 191(7.7) | 58(4.7) | 0.001 |
| Arrhythmia | 1176(31.5) | 778(31.2) | 398(31.9) | 0.654 |
| Stroke | 812(21.7) | 555(22.3) | 257(20.6) | 0.250 |
| Pulse >109 beats per min | 414(11.1) | 277(11.1) | 137(11.0) | 0.912 |
| SBP <90 mm Hg | 59(1.6) | 36(1.4) | 23(1.8) | 0.353 |
| DBP ≤60 mm Hg | 365(9.8) | 243(9.8) | 122(9.8) | 0.969 |
| Respirations≥30 per min | 70(1.9) | 45(1.8) | 25(2.0) | 0.670 |
| Altered mental status | 247(6.6) | 178(7.1) | 69(5.5) | 0.063 |
| Anemia | 1975(53.1) | 1298(52.3) | 677(54.6) | 0.189 |
| WBC >10×103 mm-3 | 1001(26.9) | 666(26.8) | 335(27.0) | 0.900 |
| ESOR<2% | 2648(71.8) | 1786(72.7) | 862(70.1) | 0.101 |
| PH <7.3 | 105(2.8) | 67(2.7) | 38(3.0) | 0.529 |
| BUN >7 mmol/L | 1610(43.1) | 1082(43.4) | 528(42.4) | 0.544 |
| NT-pro BNP>1000 pg/ml | 1668(50.7) | 1147(52.5) | 521(47.2) | 0.004 |
| Troponin T>200 ng/L | 127(4.3) | 95(4.9) | 32(3.2) | 0.036 |
| Consolidation | 1324(35.4) | 854(34.3) | 470(37.7) | 0.038 |
| Pleural effusion | 1142(30.6) | 742(29.8) | 400(32.1) | 0.145 |
| BAP-65 | 2(2-3) | 2(2-3) | 2(2-3) | 0.100 |
| CURB-65 | 1(1-2) | 1(1-2) | 1(1-2) | 0.383 |
| DECAF | 2(2-3) | 2(2-3) | 2(2-3) | 0.549 |
| NIVO | 3(2-4) | 3(2-4) | 3(2-4) | 0.207 |

Data are presented as the number of patients (%); mean ± standard deviation; median (interquartile range)

Abbreviations: CVDs=cardiovascular diseases; SBP= systolic blood pressure; DBP= diastolic blood pressure; WBC= white blood cell; EOSR=eosinophil ratio; BUN=blood urea nitrogen; NT-pro BNP=N-terminal pro-B-type natriuretic peptide;

Note: Anemia: hemoglobin is less than 12g/L in females and hemoglobin is less than 13g/L in males

Supplementary Figure S1 – A, B, Receiver operating characteristic curves of the ABCDMP, BAP-65, CURB-65, DECAF, and NIVO scores and their corresponding area under the curves: (A) derivation cohort and (B) validation cohort.


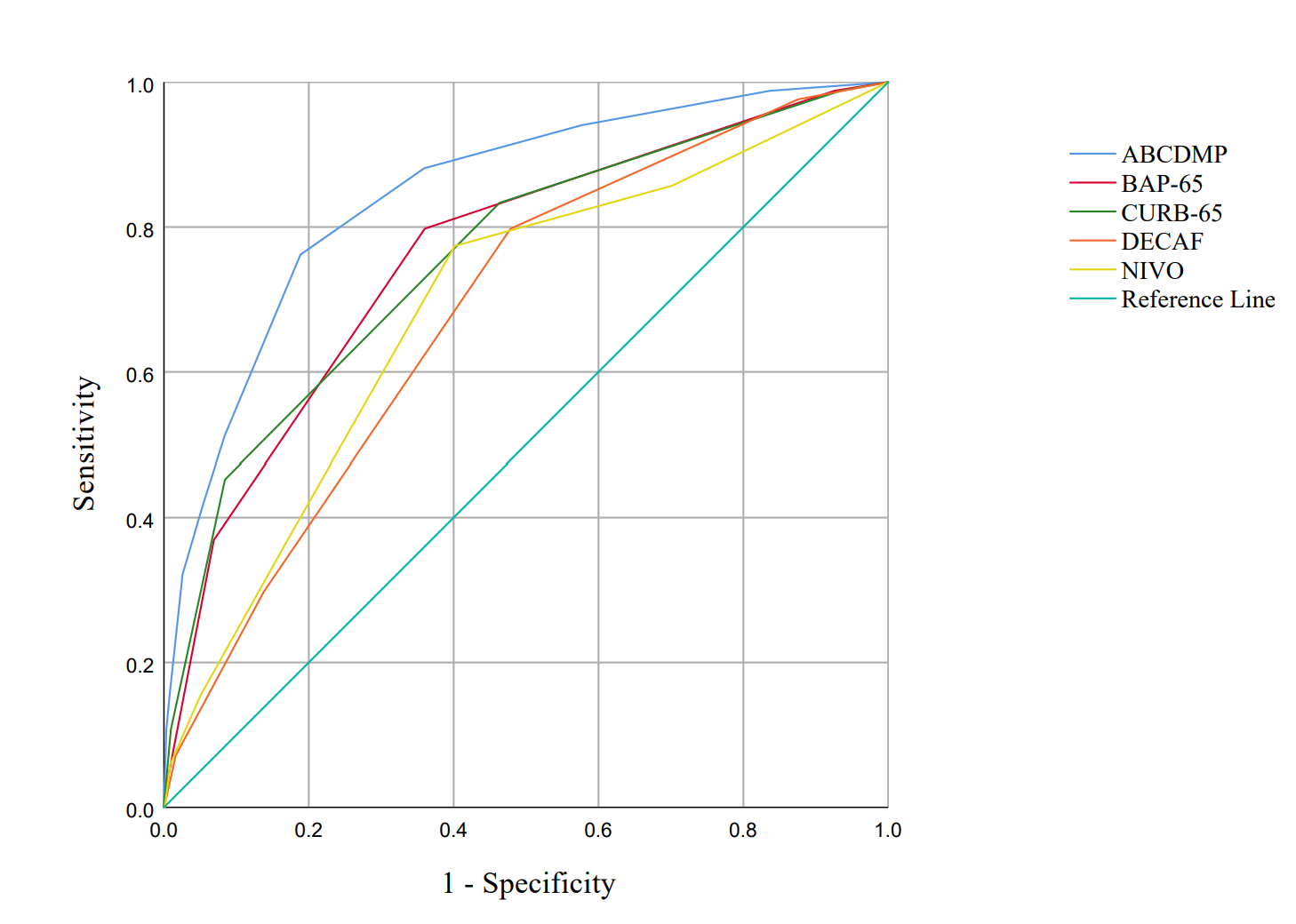


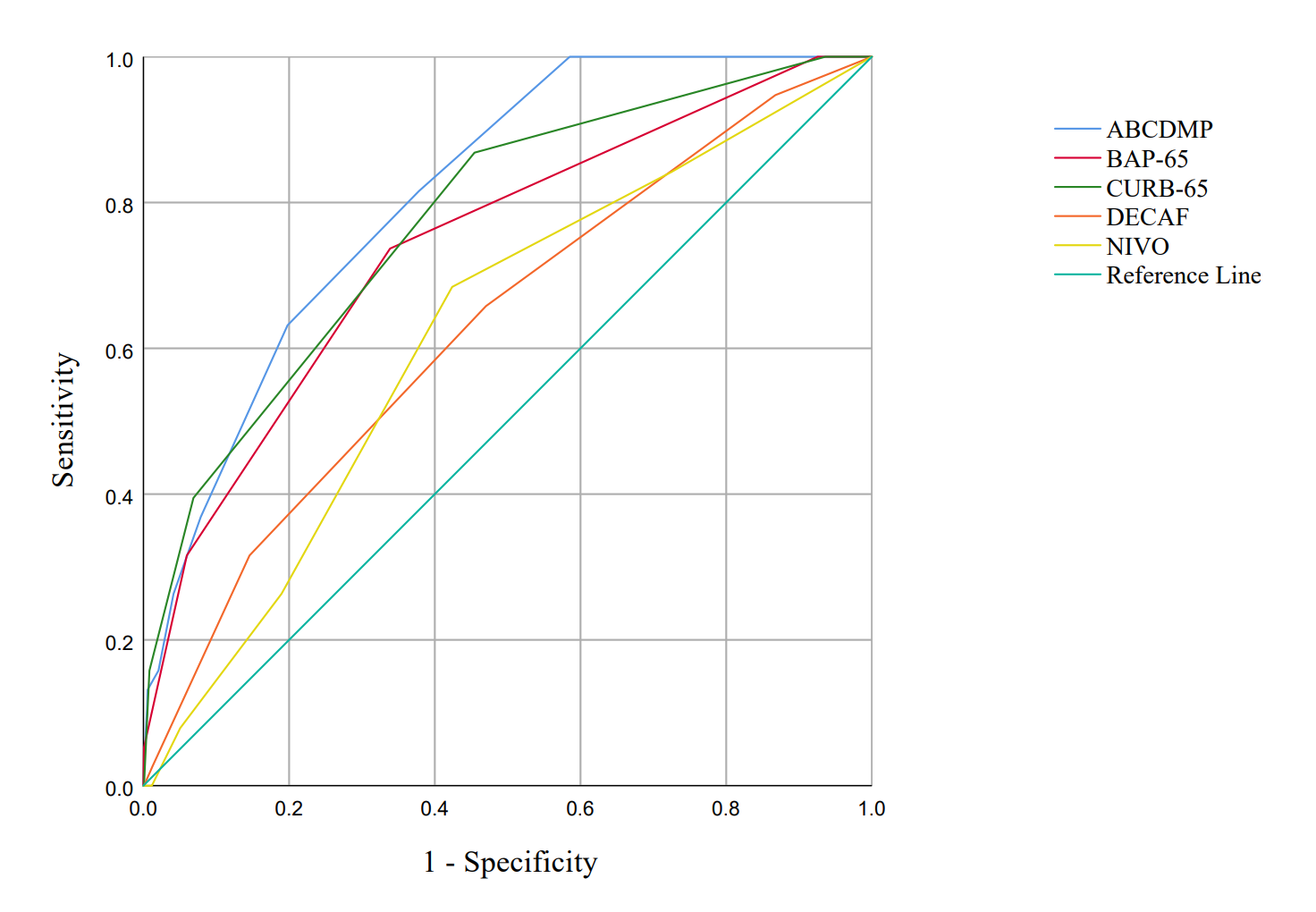


Supplementary Figure S2 Receiver operating characteristic curves for the ABCDMP score in predicting 3-year mortality in patients with AECOPD and CVDs.

(Abbreviations: AECOPD= Acute exacerbation of chronic obstructive pulmonary disease; CVDs= cardiovascular diseases)


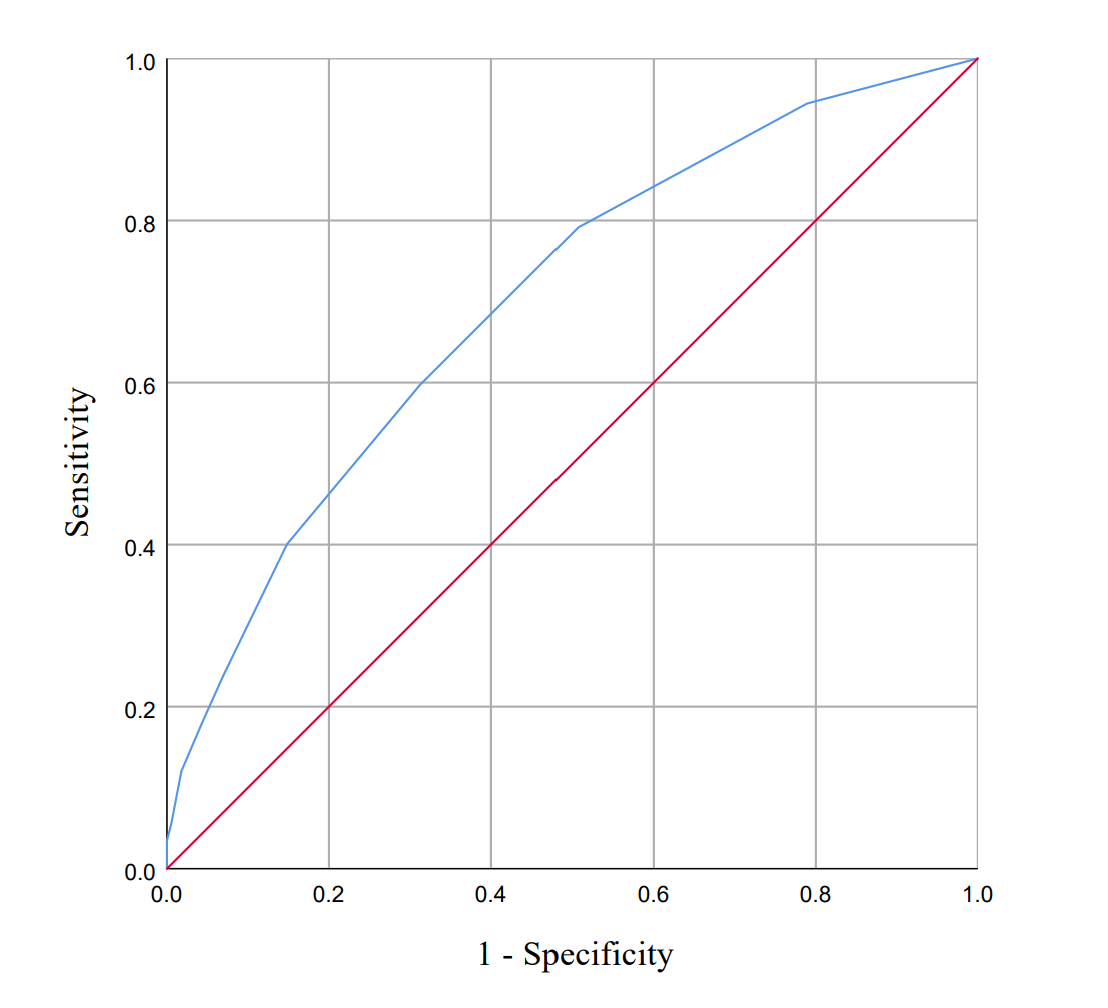

Supplement: Supplementary file 1 — Supplementary Material 1 [file 12931_2024_2704_MOESM1_ESM.docx]
